# Fatty Acid Profile of Milk and Cheese from Dairy Cows Supplemented a Diet with Palm Kernel Cake

**DOI:** 10.3390/molecules200815434

**Published:** 2015-08-24

**Authors:** Ronaldo Oliveira, Mario Faria, Raimundo Silva, Leilson Bezerra, Gleidson Carvalho, Alyson Pinheiro, Juliana Simionato, André Leão

**Affiliations:** 1 School of Veterinary and Animal Science, Federal University of Bahia, Salvador 40170110, Brazil; E-Mails: ronaldooliveira@ufba.br (R.O.); jagualyson@bol.com.br (A.P.); 2 Department of Animal Science, Federal Institute of Education, Science and Technology of Bahia, Salvador 40170110, Brazil; E-Mails: mariomarcosfaria@hotmail.com (M.F.); rluizvaz@gmail.com (R.S.); gleidsongiordano@ufba.br (G.C.); 3 Department of Animal Science, Federal University of Piauí, Bom Jesus 6490000, Brazil; 4 Department of Animal Science, State University of Bahia, Itapetinga 45700000, Brazil; E-Mail: jsimionato@utfpr.edu.br; 5 Institute of Agricultural Sciences and Technology, Federal University of de Mato Grosso, Rondonópolis 78735636, Brazil; E-Mail: agleao@yahoo.com.br

**Keywords:** atherogenicity index, CLA, *Elaeis guineensis*, fatty acid profile, unsaturated

## Abstract

Lipid supplements (oilseeds vegetables) are included in ruminant diet to increase its energy density and improve fatty acid composition of milk and consequently of fresh cheese. Milk and cheeses were evaluated from crossbred Holstein × Zebu, fed diets enriched with 0%, 25%, 50%, and 75% inclusion levels of palm kernel cake in concentrated supplement, which were supplied daily (3.0 kg). Milk and fresh cheese (*p* = 0.001) fatty acids C12:0 exhibited quadratic negative values. Milk fatty acids C13:0, C20:0, C18:2t10c12, and C20:2n-6 presented positive quadratic values. The milk C18:2n-6 decreased linearly and in fresh cheese exhibited an increasing linear effect (*p* = 0.016). However, the fatty acids grouped in milk fat were not affected. The medium-chain fatty acids varied negatively and quadratically (*p* = 0.045). There was no effect on milk and fresh cheese chemical composition (*p* > 0.05). The milk fat was increased (*p* = 0.0065) quadratically (minimum point of 24.7%). Thus, the addition of palm kernel cake to cow diets negatively altered the fatty acid profile, it raises the percentage of lauric (C12) and tridecanoic (C13) acids fat which is not beneficial to human health from a nutraceutical perspective, although it did not influence the atherogenicity index.

## 1. Introduction

Milk and dairy products are important sources of fatty acids (FA) in the human diet [1]. However, there are health concerns about high concentrations of saturated fatty acids (SFA) in milk fat. Most importantly, lauric acid (C12:0), myristic acid (C14:0), and palmitic acid (C16:0) have all been linked to negative effects on human health, especially an increased risk of cardiovascular disease, although more recent reviews recommend the main target of improving milk quality should be a decrease in C16:0, due to its relatively high concentrations in milk fat [2].

The inclusion of cakes from palm fruits in ruminant diets has attracted the attention of the scientific community and stimulated research [3,4,5] on the influence of these new feedstuffs on production and quality of milk and cheese [4,6,7]. Palm kernel cake, a byproduct of this industrial process of Oil palm cultivation (*Elaeis guineensis*), has been used in ruminant feed because it is a low cost supplement in relation to traditional ingredients (corn and soybean), and it has a reasonable total digestible nutrients concentration (65% to 70%) [8].

In this scenario, palm kernel cake, a product of dried pulp of palm oil after grinding and extraction of its oil for the production of biodiesel or for human nutrition, has been studied as an alternative feed for cattle [9,10]. This is due to the need to formulate diets to lower costs, seeking increased profitability in the production system, without compromising milk and its derivatives’ quality [11,12]. Therefore, it is extremely important to know the optimal levels of palm kernel cake included in the diet of lactating cows. Thus, it was also hypothesized that the use of palm kernel cake in the diet of lactating cows, despite the presence of short-chain fatty acids (C12 and C14), is possible without harming the profile of fatty acids of the milk and cheese produced.

Given the nutritional benefits of cow milk and its derivatives, the objective of this study was to determine the best level of palm kernel cake to add to supplements for grazing cows without harming the production, chemical composition, and fatty acid profile of the milk and fresh cheese.

## 2. Results and Discussion

### 2.1. Production and Chemical Composition of the Milk and Fresh Cheese

The dry matter intake (*p* = 0.2442), milk production (*p* = 0.2318) and corrected milk production (*p* = 0.2101) was not affected by the levels of inclusion of palm kernel cake in the dietary supplement (Table 1). Cheese yields (L/kg) (*p* = 0.8863) and adjusted for 58% moisture (*p* = 0.9220) presented same across treatment groups.

The crude protein, lactose, total solids, and nonfat dry milk solids of milk and fresh cheese were not affected (*p* ≥ 0.05) by the palm kernel cake levels in the concentrate. The cheese fat was not affected (*p* = 0.8117), however, the milk fat content increased quadratically with increases in the cake level (*p* = 0.0109), and the relation reached a minimum fat content value at a cake inclusion level of 24.7%.

**Table 1:** Means and standard errors of the dry matter intake (DMI), milk production (MP) and the production of milk corrected for 3.5% fat (MPC), fresh cheese yield (L/kg), fresh cheese adjusted yield (L/kg) and chemical composition of milk and fresh cheese produced by cows fed with different palm kernel cake levels.

Item	Level of Palm Kernel Cake (% DM)				SEM ^†^	*p*-Value *	
	0	25	50	75		Linear	Quadratic
**Milk**							
**DMI (kg/day)**	11.68	11.58	11.32	10.77	0.5661	0.2442	0.6886
**MP kg/day**	10.20	10.03	9.40	9.10	0.7166	0.2318	0.9252
**MPC ^3.5% Fat^ kg/day**	9.73	9.67	9.47	9.37	0.4290	0.2101	0.8282
**Fat (%) ^‡^**	3.3	3.2	3.4	3.9	0.0833	0.0109	0.0065
**Crude Protein (%)**	3.0	3.0	2.9	2.9	0.0189	0.0602	0.1612
**Lactose (%)**	4.6	4.4	4.5	4.6	0.0217	0.5915	0.3141
**Total Solids (%)**	11.8	11.6	11.8	12.3	0.0270	0.4098	0.0835
**NFDMS (%)**	8.5	8.4	8.4	8.4	0.0375	0.9909	0.2520
**Fresh Cheese**							
**Yield (L/kg)**	5.82	5.93	5.80	5.84	0.1492	0.8863	0.9402
**Adjusted Yield (L/kg)**	5.62	5.81	5.72	5.64	0.1857	0.9220	0.9676
**Fat (%)**	20.5	20.7	21.6	19.9	0.3402	0.8117	0.4395
**Crude Protein (%)**	17.2	17.3	17.2	17.7	0.2566	0.5964	0.8225
**Moisture (%)**	59.4	58.9	58.7	59.4	0.3217	0.9908	0.7223

* *p*-value indicates the differences between supplement levels within each feeding strategy liner or Quadratic; ^†^ Standard error of the mean; ^‡^ Ŷ = 3.334 − 0.0098TD + 0.0002 TD^2^, R^2^ = 0.99.

### 2.2. Fatty Acid Profile

Milk presented a negative quadratic relation (*p* < 0.027) between lauric acid (C12:0) and palm kernel cake levels in the supplement concentrate, with a lauric acid maximum at a cake level of 51.7% (Table 2). By contrast, positive quadratic behavior (*p* < 0.001) was observed for tridecanoic acid (C13:0), with a minimum at a concentrate cake level of 62.2%. The inclusion of palm kernel cake changed the milk levels of the following long-chain fatty acids (LCFAs): C18:2n-6 (linoleic acid), C18:2t10c12 (conjugated linoleic acid, CLA), C20:0 (arachidic), C20:2n-6 (eicosadienoic), and C20:4n-6 (arachidonic). Considering that the LCFA levels in the concentrate decreased (84%, 47%, 30% and 22%) with the increasing levels of palm kernel cake, the dietary LCFAs did not affect (*p* ≥ 0.05) the total concentrations of SCFAs, MCFAs, or LCFAs in milk to the same extent (Table 3). The C18:0 (stearic) (*p* = 0.263) and C14:1n-5 (myristoleic) (*p* = 0.241) fatty acid contents of the milk were not affected by the palm kernel cake levels.

**Table 2:** The effect of dietary supplementation with differing amounts of palm kernel cake on the fatty acid content (in g/100 g of fatty acids) of milk from cows.

Item	Level of Palm Kernel Cake (% DM)				SEM ^†^	*p*-Value *	
	0	25	50	75		Linear	Quadratic
**C10:0**	2.74	2.47	2.44	2.57	0.034	0.208	0.054
**C12:0 ^‡^**	3.43	3.96	4.23	3.98	0.008	0.027	0.009
**C13:0 ^§^**	0.08	0.08	0.07	0.07	0.001	0.001	0.000
**C14:0**	12.57	13.00	12.97	12.44	0.102	0.673	0.202
**C16:0**	32.32	32.63	32.45	32.13	0.189	0.680	0.785
**C18:0**	12.05	11.83	11.50	11.45	0.127	0.230	0.479
**C20:0**	0.47	0.41	0.42	0.47	0.008	0.877	0.012
**Other Saturated**	7.71	7.35	7.34	7.38	0.081	-	-
**C14:1n-5**	1.24	1.26	1.27	1.26	0.117	0.241	0.345
**C16:1n-7**	1.32	1.25	1.34	1.32	0.021	0.616	0.799
**C17:1n-7**	0.41	0.41	0.43	0.45	0.010	0.165	0.380
**C18:1n-7c**	0.33	0.31	0.36	0.33	0.015	0.637	0.855
**C18:1n-7t**	2.25	2.33	2.16	2.31	0.014	0.945	0.913
**C18:1n-9c**	20.38	20.08	20.54	21.14	0.267	0.572	0.760
**C18:2n-6 ^††^**	0.66	0.58	0.57	0.57	0.008	0.024	0.028
**C18:2c9t11**	0.16	0.16	0.15	0.16	0.002	0.428	0.449
C18:2t10c12 **	0.12	0.12	0.11	0.14	0.002	0.051	0.024
**C18:3n-3**	1.12	1.14	1.03	1.16	0.011	0.850	0.521
**C18:3n-6**	0.17	0.17	0.16	0.16	0.003	0.145	0.297
**C20:3n-6**	0.10	0.11	0.11	0.10	0.007	0.697	0.234
**Other Unsaturated**	1.64	1.60	1.62	1.67	0.035	-	-

* *p*-value indicates the differences between supplement levels within each feeding strategy liner or Quadratic; ^†^ Standard error of the mean; ^‡^ Ŷ = 3.424 + 0.031TD − 0.0003TD^2^, R^2^ = 0.97; ^§^ Ŷ = 0.0847 − 0.00059TD + 0.000005 TD^2^; R^2^ = 0.96; Ŷ = 0.4634 − 0.00319TD + 0.00004TD^2^; R^2^= 0.97; ^††^ Ŷ = 0.6399 − 0.0012TD; R^2^ = 0.71; ** Ŷ = 0.66 + 0.0037TD − 0.00003TD^2^; R^2^ = 0.98.

In the fresh cheese, the palm kernel cake levels in the supplement concentrate did not influence the butyric (*p* = 0.208), caproic (*p* = 0.218), caprylic (*p* = 0.292), or capric (*p* = 0.150) fatty acids (Table 3). The fresh cheeses had higher concentrations of myristic (12.44%), palmitic (31.34%), and stearic (11.41%) fatty acids and the monounsaturated fatty acid, oleic acid (21.75%), making up 76.94% of the total fatty acids present. A negative quadratic relationship between lauric acid (C12:0) (*p* = 0.001) and palm kernel cake levels in the supplement concentrate was observed, with a maximum lauric acid level at a cake level of 51.7%. By contrast, positive quadratic effect (*p* = 0.0001) was observed for tridecanoic acid (C13:0), with a minimum concentrate cake level of 62.2%.

There was no influence of supplementation on the long-chain saturated stearic fatty acid (C18:0) (*p* = 0.663). The linoleic fatty acid (*p* = 0.016) showed a linear increase with supplementation with palm kernel cake, while the fatty acid 20:2n-6 showed a negative linear (*p* = 0.004) relationship with cake supplementation.

**Table 3:** The fatty acid profile (%) of the fat from fresh cheese that was produced with milk from pastured cows receiving supplements with palm kernel cake.

Fatty Acids	Palm Kernel Cake Levels (% DM)				SEM ^†^	*p*-Value *	
	00	25	50	75		Linear	Quadratic
**C4:0**	2.86	2.36	2.52	2.66	0.072	0.569	0.208
**C6:0**	2.22	1.89	2.01	2.05	0.031	0.394	0.218
**C8:0**	1.33	1.16	1.23	1.21	0.016	0.292	0.340
**C10:0**	2.70	2.39	2.46	2.41	0.034	0.150	0.238
**C12:0 ^‡^**	3.28	4.12	4.31	4.02	0.008	0.021	0.001
**C13:0 ^§^**	0.08	0.06	0.07	0.07	0.001	0.006	0.0001
**C14:0**	12.06	12.70	12.53	12.41	0.102	0.676	0.382
**C18:0**	11.58	11.57	11.18	11.28	0.127	0.363	0.663
**Other Saturated**	33.03	34.22	33.77	33.64	0.460	-	-
**C14:1n-5**	1.17	1.20	1.24	1.04	0.017	0.142	0.028
**C16:1n-7**	1.35	1.37	1.47	1.39	0.021	0.558	0.541
**C18:1n-7c**	0.54	0.45	0.50	0.54	0.015	0.901	0.099
**C18:1n-7t**	2.17	2.54	1.76	2.06	0.014	0.068	0.186
**C18:1n-9c**	22.33	20.85	21.80	21.92	0.267	0.863	0.739
C18:2n-6 **	0.61	0.61	0.64	0.65	0.010	0.016	0.054
**C18:2c9t11**	0.16	0.16	0.15	0.14	0.002	0.065	0.184
**C18:2t10c12**	0.11	0.11	0.11	0.11	0.002	0.587	0.831
**C18:3n-3**	1.21	1.11	1.11	1.17	0.011	0.452	0.074
**C18:3n-6**	0.16	0.17	0.16	0.16	0.003	0.500	0.662
**C20:2n-6 ^††^**	0.04	0.03	0.03	0.03	0.011	0.004	0.013
**Other Unsaturated**	1.03	0.93	0.97	1.03	0.043	-	-

* *p*-value indicates the differences between supplement levels within each feeding strategy liner or Quadratic; ^†^ Standard error of the mean; ^‡^ Ŷ = 3.3026 + 0.0439PKC − 0.0005PKC^2^, R^2^ = 0.99; ^§^ Ŷ = 0.0771 − 0.00069PKC + 0.000007PKC^2^, R^2^ = 0.71; Ŷ= 1.1605 + 0.00572PKC − 0.0001PKC^2^, R^2^ = 0.88; ** Ŷ = 0.6029 + 0.0007PKC, R^2^ = 0.88; ^††^ Ŷ = 0.0381 − 0.0000000005PKC, R^2^ = 0.73.

### 2.3. Grouped Fatty Acid Profile and Atherogenicity Index (AI)

The grouped fatty acid profile (g/100 g of fatty acids) (*p* ≥ 0.05) from grazing cows were not affected by the palm kernel cake levels in the concentrate (Table 4).

However, the values of grouped fatty acids of fresh cheese (Table 5), it was verified that only PUFA (*p* = 0.036) and medium-chain fatty acids (MCFA) (*p* = 0.045) from fresh cheese differed among the treatments, varying positively (minimum point of 48.3) and negatively (maximum point of 40.6), respectively, in a quadratic manner. By evaluating the SFA (69.93%), MUFA (27.83%), and PUFA (2.34%), it was observed that there is a highly elevated amount of SFA, a medium amount of MCFA and a lower quantity of PUFA in the fresh cheese. Another factor to consider is the oxidation of the PUFA, which represented 2.37%, 2.33%, 2.16% and 2.34% of the total fatty acids in the fresh cheese and corresponded to the diets with 0%, 25%, 50% and 75% palm kernel cake in the concentrate, respectively.

The C12:0 (lauric acid) differed between treatments, but the AI verified in the fresh cheese did not vary (*p* = 0.532), because of the similarity between C14:0 (myristic) and C16:0 (palmitic) acids, which have a greater weight in the formula used to calculate this index because they are hypercholesterolemic (with an elevated atherogenic potential).

**Table 4:** The effect of dietary supplementation with differing amounts of palm kernel cake on the grouped fatty acid profile (in g 100 g^−1^ of fatty acids) of milk from grazing cows.

Item	Palm Kernel Cake Levels (%DM)				SEM ^†^	*p-*Value *	
	0	25	50	75		Linear	Quadratic
**Summations**							
Saturated	71.36	71.73	71.42	70.49	0.252	0.322	0.445
Unsaturated	28.64	28.27	28.58	29.51	0.005	0.596	0.689
Monounsaturated	26.28	25.94	26.41	27.17	0.005	0.531	0.695
Polyunsaturated	2.37	2.33	2.16	2.34	0.004	0.221	0.079
Short Chain	8.55	8.05	7.94	8.01	0.106	0.160	0.215
Medium Chain	52.48	53.59	53.79	52.76	0.265	0.878	0.326
Long Chain	38.97	38.35	38.27	39.23	0.347	0.940	0.704
CLA	0.28	0.28	0.26	0.30	0.003	0.401	0.102
**Ratios**							
Unsaturated/Saturated	0.46	0.43	0.44	0.45	0.057	0.174	0.099
*n*-6/*n*-3	0.86	0.78	0.82	0.76	0.012	0.219	0.462
18:1 *trans*-11/18:0 ^‡^	0.19	0.20	0.19	0.20	0.002	0.317	0.587
∑ *trans*/∑ C18 ^§^	2.54	2.60	2.43	2.60	0.036	0.988	0.837
AI	3.05	3.16	3.17	2.99	0.044	0.791	0.526

* *p-*value indicates the differences between supplement levels within each feeding strategy liner or Quadratic; ^†^ Standard error of the mean; ^‡^ Ratio of vaccenic to stearic acid; ^§^ Ratio of total trans fatty acids to fatty acids with 18 carbons; Atherogenicity index.

**Table 5:** The profile of grouped fatty acids (%) of fat from fresh cheese that was produced with milk from pastured cows receiving supplements with palm kernel cake.

Item	Palm Kernel Cake Levels (%DM)				SEM ^†^	*p-*Value *	
	00	25	50	75		Linear	Quadratic
**Summations**							
Saturated	69.25	70.77	70.22	69.47	0.281	0.978	0.603
Unsaturated	31.77	30.33	30.68	30.71	0.327	0.555	0.667
Monounsaturated	28.53	27.36	27.68	27.75	0.315	0.659	0.757
Polyunsaturated ^‡^	2.49	2.29	2.29	2.30	0.024	0.051	0.036
Short-Chain	9.11	7.84	8.24	8.29	0.120	0.303	0.190
Medium-Chain ^§^	50.25	53.24	52.74	51.60	0.300	0.381	0.045
Long-Chain	40.91	39.34	39.21	39.63	0.350	0.389	0.431
Total CLA	0.27	0.26	0.26	0.25	0.003	0.122	0.303
**Ratios**							
Unsaturat/Saturated	0.46	0.43	0.44	0.45	0.544	0.174	0.099
*n*-6/*n*-3	0.83	0.81	0.84	0.74	0.010	0.135	0.197
18:1 *trans*-11/18:0	0.19	0.22	0.16	0.18	0.015	0.175	0.393
∑ *trans*/∑ C18 **	2.44	2.81	2.01	2.30	0.078	0.092	0.235
AI ^††^	2.66	2.96	2.86	2.86	0.007	0.532	0.448

* *p-*value indicates the differences between supplement levels within each feeding strategy liner or Quadratic; ^†^ Standard error of the mean; ^‡^ Ŷ = 2.4836 − 0.0087PKC + 0.00009PKC^2^, R^2^ = 0.94; ^§^ Ŷ = 50.396 + 0.138PKC − 0.0017PKC^2^, R^2^ = 0.92; Ratio between vaccenic and stearic fatty acids; ** Ratio between total trans and 18-carbon fatty acids; ^††^ Atherogenicity index.

### 2.4. CLA Isomers in the Fat of Cheeses

The CLA values were not influenced (*p* = 0.303) by the cake treatments, with an average value to milk and fresh cheese of 0.28 and 0.26 g/100 g (Table 4 and Table 5). The use of milk from pastured cows supplemented with palm kernel cake in their diet concentrate did not reduce the CLA content in the cheeses.

### 2.5. Discussion

#### 2.5.1. Influence of Palm Kernel Cake on Milk and Fresh Cheese Composition 

The similarity between milk production is associated with no difference between the dry matter intakes of animals. The milk production and the milk production corrected for 0.035 fat (9.96 and 9.56 kg/day, respectively) can be considered normal for crossbred dairy herds. These values are associated with a lower degree of specialization of the experimental herd due to their low genetic potential. The increase in milk fat can be attributed to the higher content of ether extract and fiber present in palm kernel cake, observed in Table 1 and Table 2, which may have favored the acetic fermentation. Another factor that may have contributed is the largest ether extract intake [13]. Generally, additional sources of lipids added to the animal diet are especially long chain fatty acids (Table 6), which have a great influence on the increase of their concentration in milk fat, or not after suffering biohydrogenation by microbial action in the rumen. The process of saturation of fatty acids by rumen microorganisms aims to reduce its reactivity and thus protect the integrity of the microbial membrane lipoprotein. However the case of palm oil, there is a greater quantity of saturated (70%) in relation to unsaturated (30%), which is beneficial for ruminal microorganisms and can explain the similarity in the milk and cheese yield even at the level of 75% of palm kernel cake [11,14]. 

#### 2.5.2. Fatty Acid Profile

In this study, there was a linear decrease in the metabolizable energy and net energy of concentrate to lactation in cows’ supplements with levels of palm kernel cake (Table 7). Overall, there was a greater cost for the net energy of maintenance and a decreased energy balance in cows with greater amounts of palm kernel cake in their diet. However, the composition of fatty acids suggest that it is not possible to attribute these results to the influence of a single factor.

The primary source of short-chain fatty acids, such as C13:0, is volatile fatty acids, such as propionic and valeric acids. Short-chain fatty acids are incorporated into the membrane structure of bacteria at the time of food fermentation. The steps that regulate this process are not yet clear; however, bacteria may directly incorporate fatty acids from the diet (Table 6).

Milk fat has high concentrations of short chain fatty acids compared with other foods. These fatty acids have the characteristic of being volatile, which will give much of the aroma and flavor of many dairy products, mainly butter and cheeses and, in the case of cheese, these acids contribute to characterize different name brands [15]. Short chain fatty acid concentrations observed in this experiment were sufficient to overcome in concentrations often necessary to saturate the lipases present in the mass of cheese, if this milk was intended for that purpose. So, theoretically, the milk produced in this experiment does not alter the organoleptic qualities of the cheese, but sensory studies are necessary to affirm such a hypothesis.

Among the medium-chain fatty acids, special attention must be given to saturated fatty acids C16:0 (palmitic acid) and C14:0 (myristic acid). Myristic acid is the most hypercholesterolemic and has a fourfold greater potential than palmitic acid to raise the plasma cholesterol concentration. The mean values observed in milk for increasing levels of palm kernel cake were 13% for myristic acid and 32% for palmitic acid, which exceeded the means observed by Moate *et al*. [16] of 10% and 28.5% for the respective fatty acids.

High concentrations of saturated MCFA (lauric, myristic, and palmitic) in the palm kernel cake have promoted increases in these fatty acids in diets that were offered (Table 6) and consequently in milk and cheese that were produced by cows (Table 1). And the increase in palmitic acid (C16:0) occurred due to the large amount contained in Massai grass. Therefore, how all experimental groups received pasture will explain the similarity between C16 concentrations in milk (Table 2). Despite the increase in lauric and myristic acid, the quantities were not sufficient to change the milk (*p* = 0.791) and fresh cheese (*p* = 0.532) Atherogenicity index (AI).

**Table 6:** Fatty acid compositions of the palm kernel cake, concentrate supplements, and Massai grass when expressed as a percentage of the total fatty acids.

Fatty Acid		Palm Kernel Cake	Palm Kernel Cake Levels(%DM)				Massai Grass
			0	25	50	75	
C6:0	(caproic)	0.30	0.02	0.18	0.33	0.32	0.00
C8:0	(caprylic)	3.77	0.05	2.43	4.88	5.38	0.00
C10:0	(capric)	3.48	0.06	2.16	3.89	4.34	0.00
C12:0	(lauric)	47.40	0.46	24.44	39.81	45.48	0.00
C13:0	(tridecanoic)	0.05	0.00	0.03	0.03	0.04	0.00
C14:0	(myristic)	16.66	0.30	8.53	12.45	14.61	4.29
C15:0	(pentadecanoic)	0.05	0.03	0.03	0.04	0.05	0.56
C16:0	(palmitic)	7.99	12.49	9.23	7.53	7.67	41.82
C17:0	(heptadecanoic)	0.06	0.07	0.03	0.02	0.01	0.00
C18:0	(stearic)	2.85	2.23	4.15	2.61	2.76	6.18
C20:0	(arachidic)	0.06	0.06	0.02	0.28	0.05	0.00
C14:1n-5	(myristoleic)	0.02	0.01	0.03	0.04	0.05	0.37
C15:1n-5	(pentadecenoic)	0.00	0.00	0.02	0.02	0.02	0.00
C16:1n-7	(palmitoleic)	0.04	0.17	0.07	0.03	0.03	0.00
C17:1n-7	(heptadecenoic)	0.02	0.04	0.00	0.00	0.00	0.00
C18:1n-7t	(trans-vaccenic)	0.03	0.20	0.44	0.68	0.61	0.00
C18:1n-9c	(oleic)	13.84	34.74	22.42	15.10	13.72	6.75
C18:1n-7c	(*cis*-vaccenic)	0.04	1.37	0.84	1.00	0.03	0.00
C18:2n-6	(linoleic)	2.64	45.04	17.90	8.03	5.04	9.41
C18:3n-3	(α-linolenic)	0.00	0.00	0.00	0.00	0.00	29.95
C18:3n-6	(γ-linolenic)	0.13	0.48	0.30	0.16	0.15	0.00
C18:2c9t11	(rumenic)	0.07	0.24	1.19	2.03	0.09	0.00
C20:3n-6	(eicosatrienoic)	0.06	0.27	0.17	0.12	0.06	0.02
C20:4n-6	(arachidonic)	0.09	0.08	0.05	0.03	0.03	0.00

**Table 7:** Proportion of ingredients and chemical composition of the concentrates used to supplement lactating cows when calculated as percentages on a dry matter basis.

Food Ingredient	Palm Kernel Cake Levels (%DM)			
	0	25	50	75
**Proportion of Ingredients (g/kg DM)**				
Ground Corn	801.7	596.6	391.4	186.3
Soybean Meal	139.9	95.0	50.2	05.3
Palm Kernel Cake	00.0	250.0	500.0	750.0
Mineral Mixture	30.0	30.0	30.0	30.0
Urea	25.7	25.7	25.7	25.7
Ammonium Sulfate	02.7	02.7	02.7	02.7
**Chemical Fraction (g/kg DM)**				
Dry Matter	898.6	908.8	916.9	925.3
Organic Matter	984.8	988.8	992.8	996.8
Mineral Matter	15.2	11.2	07.2	03.2
Crude Protein	199.6	188.6	177.1	165.6
Ether Extract	18.1	43.6	69.1	94.5
Neutral Detergent Fiber	107.9	257.3	406.5	555.7
Non-Fibrous Carbohydrate	659.2	499.3	340.1	181.0
Total Carbohydrate	767.1	756.6	746.6	736.7
Total Digestible Nutrients ^†^	787.4	755.0	720.7	686.7
Metabolizable Energy (Mcal/kg) ^†^	34.0	30.0	26.0	25.5
Net Energy for Lactation (Mcal/kg) ^†^	22.0	19.2	16.4	16.0

^†^ Values estimated by the National Research Council formula (NRC, 2001).

A mild alteration in the long chain fatty acids (C18:2n-6 and C18:2t10c12) and cheese (C18:2n-6 and C20:2n-6) was due to the presence of oleic fatty acid (FA 13.84 total) and linoleic (2.64% total FA) on the log of palm kernel cake. Although stearic acid is the primary product of UFA ruminal biohydrogenation, several factors affect this process, such as the lipid content of the diet and the ruminal pH. When the last biohydrogenation step is inhibited, there is an accumulation of trans C18:1, 11t fatty acids [17], which are formed mostly from vaccenic acid, a product of C18:2n-6 (linoleic) biohydrogenation. Therefore, it is possible that the presence of palm kernel cake in the diet inhibited biohydrogenation and, consequently, FA passing intermediates for absorption.

The percentage of heneicosanoic acid of fresh cheese in relation to the total profile was notably minimal (0.01%), so it must not be considered a potential modifier of the fatty acid profile of these products, given its inexpressive variation.

The palm kernel cake also had a high content of unidentified fatty acids (Table 2), probably due to the fact that unsaturated fatty acids of palm kernel cake are available for biohydrogenation providing production to numerous isomeric forms [18], which were not determined in this study.

Given the similarity of the ratio of vaccenic to stearic fatty acids (Vac/18:0) and total trans to 18-carbon fatty acids (Total trans/c18), we can infer that the all treatments promoted a similar biohydrogenation process. Thus, considering that the animals were supplemented on pasture, both the biohydrogenation and enzymatic activity of Δ-9 desaturase in the mammary gland were similar across all treatments in the present study. Another important result, which is concomitant to these findings, is that the CLA values were not influenced by the cake treatments, with an average value to milk and fresh cheese of 0.28 and 0.26 g/100 g (Table 4 and Table 5). The composition of the CLA mixture (C18:2t10c12 and other isomers) varies with the diet that the animals are consuming [19]. Using concentrates in the diet promotes a change in the fatty acid profile because of microbial fermentation. 

## 3. Experimental Section 

### 3.1. Ethical Considerations

This study was carried out in strict accordance with the recommendations in the Guide for the National Council for Animal Experiments Control (NCAEC). The protocol was approved by the Committee on the Ethics of Animal Experiments of the Federal University of Bahia, Salvador, BA, Brazil (Permit Number: 17-2014).

### 3.2. Experimental Design, Animals, and Feeding

The experiment was conducted at the experimental farm of the School of Veterinary Medicine and Animal Science for the Federal University of Bahia. The farm is located in the municipality of São Gonçalo dos Campos, Salvador, BA, Brazil at a latitude of 12°23′58′′ south and a longitude of 38°52′44′′ west in the mesoregion of north central Bahia and the micro region of Feira de Santana, Salvador, BA, Brazil.

The study employed 16 multiparous crossbred Holstein × Zebu cows with an average weight of 436.6 kg (±59.7), 60–90 days postpartum, at the peak of lactation. Ten days before the first adaptation period (described below), the experimental herd was dewormed (1% ivermectin) and supplemented with an injectable solution containing vitamins A, D, and E (Adethor, Tortuga, Brazil) together with a mixture of mineral salts, B complex vitamins, and amino acids (Mod Plus). The experiment lasted 60 days and was divided into four periods of 15 days, with each consisting of 11 days of adaptation and four days of sample collection. The cows were allocated in four groups that were tested simultaneously by using a 4 × 4 Latin square design (four treatments × four periods × four animals in each treatment). 

The animals were managed in seven Massai grass (*Panicum maximum* cv. Massai) paddocks, which covered an area of 1.93 ha and were surrounded by electric fencing, under rotational grazing and variable stocking with five days of occupation and 35-days of rest. All the paddocks had shaded areas and an ad libitum supply of water and mineral supplements. “Regulator” animals were used to adjust the available feed supply to 8% of the body weight in dry matter (DM) when necessary. The concentrate supplement was offered at 3.0 kg per day and divided into two daily feedings at 6:00 h and 15:00 h.

The foods used as ingredients in the four concentrate supplements included the following: palm kernel cake, soybean meal, ground corn, mineral mixture, urea, and ammonium sulfate (Table 7). The palm kernel cake was incorporated into the supplements at 0%, 25%, 50% and 75% levels on a DM basis.

Samples of feeds and feces were pre-dried at 55 °C for 72 h, ground with a Willey mill (Tecnal, Piracicaba, São Paulo, Brazil) with a 1 mm sieve, stored in air tight plastic containers (ASS, Ribeirão Preto, São Paulo, Brazil), and sealed properly until the laboratory analysis chemical composition according to AOAC [20]: levels of the dry matter (DM) (Method 967—AOAC, 1990), ash (Method 942—AOAC, 1990), crude protein (CP) (Method 981.10—AOAC, 1990), and ether extract (EE) (Method 920—AOAC, 1990). To determine the neutral detergent fiber (NDF) and acid detergent fiber (ADF) contents, the methodology of Van Soest *et al.* [21] was used with the modifications that were proposed in the Ankom device manual (Ankom Technology Corporation, Macedon, NY, USA). Acid detergent lignin (ADL) was determined according to method 973.18 of AOAC [22], in which the ADF residue was treated with 72% sulfuric acid. The non-fibrous carbohydrates (NFC) were calculated according to Mertens [23], and the neutral detergent insoluble nitrogen (NDIN) and acid detergent insoluble nitrogen (ADIN) levels were obtained according to Pereira and Rossi [24]. The levels of total digestible nutrients (TDN), metabolizable energy, and net energy for lactation were calculated according to the estimation formulae proposed by the National Research Council (NRC) [25].

### 3.3. Milk Sampling, Manufacture of Fresh Cheeses 

The animals were milked with a milking machine twice daily (at 6 h and 16 h); during these times, the dietary supplement was provided. All of the milk that was obtained from each animal was weighed and then summed to provide a daily total (milking × 2).

Milk samples were mixed in a composite sample and stored in a freezer (−20 °C), pasteurized, and processed at the end of each experimental period in the Food Technology Laboratory of the city of Feira de Santana, Bahia State, Brazil, in the dairy sector. A slow pasteurization (65 °C 30 min^−1^) method was used, and its efficiency was evaluated by alkaline phosphatase and peroxidase tests with the aid of a Laborclin kit^®^.

The manufacture of fresh cheese was performed in accordance with the flowchart suggested by Furtado [26], and the cheese yield expressed in liters of milk per kg of fresh cheese (L/kg) was calculated by the division of the total milk volume (L) by the total weight of the fresh cheese (kg) after 24 of cooling at 5 °C. The yield was adjusted for 58% uniformity by using the following formula, as described: Adjusted Yield (ADY) = (Yield) × (100—% Actual Moisture)/100 − (Desired Moisture) [26].

To calculate the milk production corrected for 0.035 fat (PMC), a formula cited by Leiva *et al.* [27] was utilized: PMC = (12.82 × Pfat) + (7.13 × Pprot) + (0.323 × PM), in which PM = production of milk, kg/day; Pfat = production of fat, kg/day; and Pprot = production of protein, kg/day. The fat content of the milk and the total nitrogen content of the milk was obtained by AOAC [20]. 

### 3.4. Chemical Composition of Milk and Fresh Cheeses

At the end of each sampling period, approximately 100 mL was preserved with Bronopol to determine the pH and chemical composition. To determine the chemical composition, the crude protein, fat, lactose, total solids, and nonfat dry extract were assessed by Milkoscan FT+ on the basis of a Fourier transform infrared analysis.

### 3.5. Milk and Fresh Cheese Fatty Acid Analysis

Milk and fresh cheese samples taken from each animal during each experimental period were stored frozen (−20 °C) until analysis to determine the fatty acid profile of the milk fat. After slow thawing, 30-mL aliquots of the milk samples were centrifuged at 12,000 rpm at 4 °C for 30 min (Hitachi Himac CR 21E) in the Virology Laboratory at the Institute of Health Sciences, Federal University of Bahia. The supernatant, which consisted of the cream, was removed after centrifugation with a spatula and placed in 1.5-mL microtubes, and the tubes were properly labeled and immediately stored in the freezer (−20 °C). The lipids were extracted with an organic solvent mixture (chloroform and methanol) according to Folch *et al.* [28] and then treated to prepare fatty acid methyl esters in accordance with Bannon *et al.* [29] and modified with 0.25 mol/L sodium methoxide basic solution in methanol-diethyl ether (1:1). The fatty acid profile was determined by gas chromatography Thermo Finnigan Trace GC Ultra-type equipped with Ionization Detector Call (IDC) by using a 120-m fused silica capillary column (BPx70 SGE); nitrogen carrier gas (6.5 mL/min); a flame ionization detector (FID); injector and detector temperatures of 250 °C and 280 °C, respectively; and a sample injection ratio of 90:10. The run protocol for the fatty acids from the milk fat began with a 10 min period at 140 °C followed by 15 °C/min increases up to 200 °C, which was maintained for an additional minute. The temperature was subsequently increased by 10 °C/min up to 230 °C, maintained for one minute, increased by 0.4 °C/min up to 233 °C, and held constant for three minutes. Finally, the temperature was increased by 0.5 °C/min up to 238 °C for a total analysis time of 39.5 min. Peaks of fatty acids were identified by comparisson to their retention time, using one-mixing standards of FAME (Sigma, St. Louis, MO, USA). The quantification of fatty acids was made using correction factors for the peak areas, as calculated from mixtures of standard fatty acids. Fatty acids were grouped into fatty acids Short chain (SCFA—up to 10 carbons), fatty acids medium chain (MCFA—from 11 to 16 carbons), fatty acids and long chain (LCFA—above 16 carbons).

### 3.6. Atherogenicity Index (AI)

The atherogenicity index (AI), which relates the fatty acid profile to potential cardiovascular disorders, was calculated from the milk fatty acid profile according to Ulbricht and Southgate [30] by using the following equation: AI = [C12:0 + (C14:0 × 4) + C16:0]/(total unsaturated fatty acids), where, C12 = the percentage of lauric acid relative to the total fatty acids, C14 = the percentage of myristic acid relative to the total fatty acids, and C16 = the percentage of palmitic acid relative to the total fatty acids. In addition to the AI, the following parameters were determined to characterize the fatty acid profile of each supplement in the concentrate: saturated fatty acids (SFA), unsaturated fatty acids (UFA), monounsaturated fatty acids (MUFA), and polyunsaturated fatty acids (PUFA); short-chain fatty acids (SCFA), medium-chain fatty acids (MCFA), and long-chain fatty acids (LCFA); the ratios between UFA and SFA; the ratio of omega-6 (n-6) to omega-3 (n-3) fatty acids; the ratio of vaccenic (C18:1) to stearic (C18:0) acid; and the ratio of total trans fatty acids to fatty acids with 18 carbons (C18).

### 3.7. Statistical Analysis 

The statistical model included the fixed effects, the level of inclusion of palm kernel cake (0%, 25%, 50% and 75%) and random effects of period (4), animals (16), and residue. We have used milk production as covariate. The data were subjected to an analysis of variance and regression analysis (linear and quadratic) by using the GLM and REG procedures of SAS^®^ statistical software (version 9.1.2. Cary, NC, USA) [31]. The significance level was set at a probability of 5% (*p* ≤ 0.05).

## 4. Conclusions

Lipid supplementation with palm kernel cake in the diet of dairy cows did not change the milk production, yield, chemical composition, and total fat content of milk and fresh cheese; however, supplementation promoted an increase in the percentage of saturated fatty acids C12:0, C13:0 and not altered percentage of PUFA, LCFA, CLA, and AI. Therefore, the use of palm kernel cake in lactating cows’ diet to the level of 75% is a low-cost alternative for lactating cows’ diets.
